# High mobility group box protein 1 in complex with lipopolysaccharide or IL-1 promotes an increased inflammatory phenotype in synovial fibroblasts

**DOI:** 10.1186/ar3450

**Published:** 2011-08-26

**Authors:** Heidi Wähämaa, Hanna Schierbeck, Hulda S Hreggvidsdottir, Karin Palmblad, Anne-Charlotte Aveberger, Ulf Andersson, Helena Erlandsson Harris

**Affiliations:** 1 Department of Women's and Children's Health, Pediatric Rheumatology Research Unit Karolinska Institutet, Astrid Lindgren Children Hospital/Karolinska University Hospital, Stockholm, 17176, Sweden; 2 Department of Medicine, Rheumatology Research Unit Karolinska Institutet, CMM Karolinska University Hospital, Stockholm, 17176, Sweden

## Abstract

**Introduction:**

In addition to its direct proinflammatory activity, extracellular high mobility group box protein 1 (HMGB1) can strongly enhance the cytokine response evoked by other proinflammatory molecules, such as lipopolysaccharide (LPS), CpG-DNA and IL-1β, through the formation of complexes. Extracellular HMGB1 is abundant in arthritic joint tissue where it is suggested to promote inflammation as intra-articular injections of HMGB1 induce synovitis in mice and HMGB1 neutralizing therapy suppresses development of experimental arthritis. The aim of this study was to determine whether HMGB1 in complex with LPS, interleukin (IL)-1α or IL-1β has enhancing effects on the production of proinflammatory mediators by rheumatoid arthritis synovial fibroblasts (RASF) and osteoarthritis synovial fibroblasts (OASF). Furthermore, we examined the toll-like receptor (TLR) 4 and IL-1RI requirement for the cytokine-enhancing effects of the investigated HMGB1-ligand complexes.

**Methods:**

Synovial fibroblasts obtained from rheumatoid arthritis (RA) and osteoarthritis (OA) patients were stimulated with HMGB1 alone or in complex with LPS, IL-1α or IL-1β. Tumour necrosis factor (TNF) production was determined by enzyme-linked immunospot assay (ELISPOT) assessment. Levels of IL-10, IL-1-β, IL-6 and IL-8 were measured using Cytokine Bead Array and matrix metalloproteinase (MMP) 3 production was determined by ELISA.

**Results:**

Stimulation with HMGB1 in complex with LPS, IL-1α or IL-1β enhanced production of TNF, IL-6 and IL-8. HMGB1 in complex with IL-1β increased MMP production from both RASF and OASF. The cytokine production was inhibited by specific receptor blockade using detoxified LPS or IL-1 receptor antagonist, indicating that the synergistic effects were mediated through the partner ligand-reciprocal receptors TLR4 and IL-1RI, respectively.

**Conclusions:**

HMGB1 in complex with LPS, IL-1α or IL-1β boosted proinflammatory cytokine- and MMP production in synovial fibroblasts from RA and OA patients. A mechanism for the pathogenic role of HMGB1 in arthritis could thus be through enhancement of inflammatory and destructive mechanisms induced by other proinflammatory mediators present in the arthritic joint.

## Introduction

The highly conserved protein high mobility group box protein 1 (HMGB1) exerts vital functions in the nucleus of all eukaryotic cells. When tissue injury is inflicted and inflammation is induced, HMGB1 can be released extracellularly and can then convey inflammatory functions. Extracellular HMGB1 may induce cytokine production, up-regulation of adhesion molecules on endothelial cells and activation of dendritic cells and T cells [1-11]. The reported presence of extracellular HMGB1 in multiple inflammatory conditions and the beneficial effects of HMGB1 blockade in preclinical models of inflammatory diseases have thus led to the acknowledgement of HMGB1 as an inflammatory mediator with pathogenic functions in several inflammatory diseases (reviewed in [12]).

HMGB1 interacts with the receptor for advanced glycated end products (RAGE), Toll-like receptor (TLR) 2 and with the TLR4 signalling complex. All three receptors are known to be involved in inflammatory processes and to possess the ability to activate NFκB translocation. RAGE-HMGB1 interaction has mainly been studied regarding induction of cell migration while HMGB1 interaction with TLR2 and TLR4 mediates immune activation. We recently reported that HMGB1-induced cytokine production in macrophages is mediated via TLR4 and requires a reduced cysteine with a thiol group in amino acid position 106, supplementing the findings of Kazama *et al*. that HMGB1 released from apoptotic cells contains an oxidized cysteine in position 106 that induces tolerance rather than immune activation [13,14].

A second mechanism for the proinflammatory function of HMGB1 is due to the ability of HMGB1 to form complexes with inflammation-inducing agents such as LPS, IL-1β, CpG-DNA (short single-stranded synthetic DNA molecules that contain a cytosine followed by a guanine) and the TLR2-ligand Pam_3_CSK_4_. Such complexes have been demonstrated to strongly enhance cytokine production in cell cultures. Additionally, in an experimental model of systemic lupus erythematosus HMGB1 was detected in circulating nucleosome complexes and the necessity of HMGB1 for these complexes to be immunogenic and to induce production of anti-DNA antibodies were demonstrated [15-20]. The molecular mechanism underlying the inflammatory activity of HMGB1 complexes and their ability to induce an enhanced response as compared to the partner molecule alone has not previously been addressed. Interestingly, it appears to be independent of the HMGB1 redox status as HMGB1, unable to induce cytokine production *per se*, still has the ability to induce such enhancement.

We and others have demonstrated an extracellular expression of HMGB1 in synovial tissue biopsies from rheumatoid arthritis (RA) patients and in joints from mice and rats with adjuvant-induced arthritis or collagen type II-induced arthritis [21-24]. Additionally, extranuclear HMGB1 localisation has been described in synovial tissue from osteoarthritis (OA) patients and in bovine osteoarthritic cartilage specimens [25,26]. Evidence for an active role of HMGB1 in arthritis pathogenesis is provided by studies demonstrating that a single injection of recombinant HMGB1 into knee joints of mice induces chronic synovitis [27] and, conversely, neutralisation of HMGB1 by treatment with antibodies or with a specific HMGB1 peptide antagonist significantly suppresses arthritis development in several studies [24,28-31].

Synovial fibroblasts (SFs) have been demonstrated to play a central role in arthritis pathogenesis, promoting both inflammation and bone and cartilage destruction [32,33]. SFs display an activated phenotype with up-regulated expression of multiple TLRs and interleukin 1 receptor type I (IL-1RI) [34-37].

We investigated whether the arthritogenic properties of HMGB1 could involve stimulation of SFs by HMGB1 complexes. We chose to study complexes formed by HMGB1 and endogenous mediators already described to be present in arthritic joints, that is, IL-1α and IL-1β, and with LPS which may also appear in arthritic joints [23,38-42]. We could demonstrate that SFs obtained from RA or OA patients responded to HMGB1 in complex with IL-1α, IL-1β or LPS, respectively, with enhanced production of tumor necrosis factor (TNF), IL-1, IL-6, IL-8 and MMP-3 and that the enhancement was mediated by interaction with IL-1RI or with TLR4, respectively. Knowing that uncomplexed HMGB1, depending on its redox status may or may not stimulate cytokine production, we initially tested the suitability of various HMGB1 batches for the present studies. We observed that every tested HMGB1 preparation, regardless of its inherent function to stimulate cytokine production, was capable to act in synergy in complexes with either LPS or IL-1α or β. In order to facilitate the read-out of the HMGB1-complex experiments we thus chose to base our studies on HMGB1 batches that did not induce cytokine formation *per se*. These experiments have enabled us to propose a mechanism by which HMGB1 contributes to both inflammatory and destructive processes activated during arthritis.

## Materials and methods

### Cell cultures

Synovial fibroblasts obtained from nine RA and six OA patients were purchased from Asterand, (Detroit, MI, USA) or propagated from synovial tissues from RA and OA patients undergoing joint replacement surgery [43]. Briefly, synovial tissues were minced and explants were maintained in DMEM supplemented with 10% heat inactivated FCS (PAA Laboratories, Linz, Austria), 100 U/ml penicillin, 100 μg/ml streptomycin and HEPES (Life Technologies, Paisely, Scotland, UK) (complete DMEM) in a tissue culture incubator at 37°C with 5% CO_2 _content. After one to two weeks of culture the tissue specimens and non-adherent cells were discarded and cells were trypsinized with Trypsin-EDTA (Gibco, Scotland, UK) and subcultured by trypsination three to four weeks after initial explantation (at 80% confluence). All SF were used for experiments between passages 3 to 8. This study was approved by the Institutional Ethical Committee (Solna, Stockholm, Sweden; ethical number 2009/1262-31/3) and is in compliance with all ethical standards and patients' consent according to the Declaration of Helsinki.

### Preparation of rHMGB1 from *E. coli*

Recombinant rat HMGB1 (rHMGB1) with a 99% identity to human HMGB1 [44] and containing a calmodulin-binding protein tag was expressed in *E. coli *strain BL21 (for sequence see ref [45]). Protein was purified by sequential ion exchange chromatography (MonoS 5/50 GL column, GE Healthcare, Chalfont St. Giles, UK) and calmodulin affinity chromatography (Calmodulin sepharose 4B, GE Healthcare). Endotoxin was removed by filtration through Acodisc Units with Mustang E Membranes (0.25 μm, Pall Life Sciences, East Hills, NY, USA), yielding endotoxin levels below 0.03 EU/μg protein as measured by the Limulus assay. Preparations of HMGB1 in 20 mM 3-(N-Morpholino) propanesulfonic acid (MOPS), 400 mM NaCl, 20 mM EGTA, 10 mM dithiothreitol at pH 8.0 were stored at -80°C until the day of use. The HMGB1 used in the studies could not induce cytokine production *per se*.

### Immunocytochemistry; TLR4 and IL-1RI expression in synovial fibroblasts

Cells were cultured on 8-well culture slides, formaldehyde-fixed and subsequently stained for the presence of TLR4 and IL-1RI as previously described [30]. Briefly, slides were incubated with 2% fetal calf sera for 10 minutes and thereafter incubated overnight with anti-TLR4 antibody (sc-8694 Santa Cruz Biotechnology Inc, Santa Cruz, CA, USA) or monoclonal rabbit anti-IL-1RI (Epitomics, Burlingame, CA, USA). Subsequently, cells were incubated with Alexa Fluor^© ^594-conjugated anti-goat or rabbit antibodies (Molecular Probes, Invitrogen, Eugene, OR, USA) and counterstained with Hoechst 33342. PBS supplemented with 0.1% saponin was used in all steps of the staining procedure. In order to verify the staining specificity, parallel blocking experiments involving preabsorption of the specific primary antibody with blocking peptide or using a primary isotype-matched irrelevant IgG were performed.

### Preparation of HMGB1-LPS, HMGB1-IL-1α and HMGB1-IL-1β complexes

HMGB1 diluted in PBS was incubated with IL-1α, IL-1β (R&D Systems, Minneapolis, MN, USA) or LPS (L-6529 Sigma, Saint Louis, MO, USA), respectively, in different ratios to give the indicated final concentrations in cell cultures. Solutions were incubated at 4°C for 16 h before addition to cell cultures. Formation of complexes has been previously demonstrated [17,18].

### TNF Elispot assay

TNF Elispot assay (Enzyme-linked immunospot assay, R&D Systems, Minneapolis, MN, USA) was performed according to the manufacturer's instructions. Briefly, Multiscreen 96-well HTS Plate Clear (MSIPS4510, Millipore, Stockholm, Sweden) were pre-wetted with 35% ethanol, washed and coated with capture antibody (Gibco, Scotland, UK) overnight. After washing, plates were blocked with cell-specific medium for 2 h in a tissue culture incubator.

Synovial fibroblasts grown to confluence were trypsinized with Trypsin-EDTA and washed with complete DMEM. Cell viability was assessed using Trypan blue (Merck, Darmstadt, Germany) exclusion in every experimental set up and determined to be 95 to 100%.

Cells were plated at 4,000 cells/well and allowed to rest for 15 to 17 h in a tissue culture incubator at 37°C with 5% CO_2 _content. Medium was discarded and cells were washed twice with OPTIMEM (Gibco, Scotland, UK) supplemented with 100 U/ml penicillin, 100 μg/ml streptomycin and stimulated for 9 h in OPTIMEM with 4 μg/ml or 100 ng/ml rHMGB1 alone or together (in complex or separately) with 1 to 100 ng/ml LPS or 0.05 to 0.5 ng/ml rIL-1β as indicated. In some experiments, cells were pre-treated for 1 to 2 h with 0.5 to 5 μg/ml IL-1RA, anakinra (Kineret; Amgen Europe, Breda, The Netherlands) or 10 μg/ml detoxified LPS L-9023 (Sigma, Saint Louis, MO, USA). Following this stimulation plates were placed on ice for 15 minutes, washed with PBS/0.05% Tween 20 (PBS/Tw) and biotinylated TNF detection antibody was added. After overnight incubation plates were washed and incubated with Streptavidine-HRP (Mabtech AB, Stockholm, Sweden).

Spots were visualized following addition of tetramethylbenzidine (TMB) chromogen liquid substrate (Mabtech) and analyzed using an AID EliSpot Reader System, (AID, Strassberg, Germany).

### Cytometric bead array (CBA) for detection of cytokine production

Cells were harvested as described for the TNF Elispot assay and 1 ml of 8 × 10^4 ^cells/ml in complete DMEM were plated in 12-well plates and rested for 15 to 17 h. Medium was discarded and cells were washed with OPTIMEM supplemented with 100 U/ml penicillin and 100 μg/ml streptomycin and stimulated as indicated with 4 μg/ml or 100 ng/ml rHMGB1 alone or in complex with 1 to 100 ng/ml LPS or 0.05 to 0.5 ng/ml, rIL-1α or rIL-1β, respectively. In some experiments cells were pre-treated for 1 to 2 h with 0.5 to 5 μg/ml IL-1RA anakinra or 10 μg/ml detoxified LPS L-9023. Supernatants were collected after 24 h of stimulation and stored at -20°C until analysis. Cell viability was assessed using Trypan blue (Merck, Darmstadt, Germany) exclusion in every experimental set up, at the beginning and at the end of every experiment and determined to be 95 to 100%.

Proinflammatory cytokine production was determined using flow CBA (B&D Biosciences, Pharmingen, San Diego, CA, USA) and analyzed according to the manufacturer's instructions.

### ELISA assay for detection of MMP-3

Cells were cultured and stimulated as described above and supernatants collected after 24 h. The release of MMP-3 was analysed by ELISA (R&D Systems, Minneapolis, MN, USA) according to the manufacturer's instruction.

### Statistical analysis

Kruskal-Wallis non-parametric ANOVA, Wilcoxon paired test or Mann Whitney were used to test statistical significance. All pair-wise comparisons were adjusted using Dunn's Multiple Comparisons Test. A *P*-value below 0.05 was considered to be statistically significant. The computer software program GraphPad Prism version 5 for Windows (GraphPad Software, San Diego, CA, USA) was used for all statistical tests.

## Results

### TLR4 and IL-1RI are expressed by synovial fibroblasts

**Figure 1:**
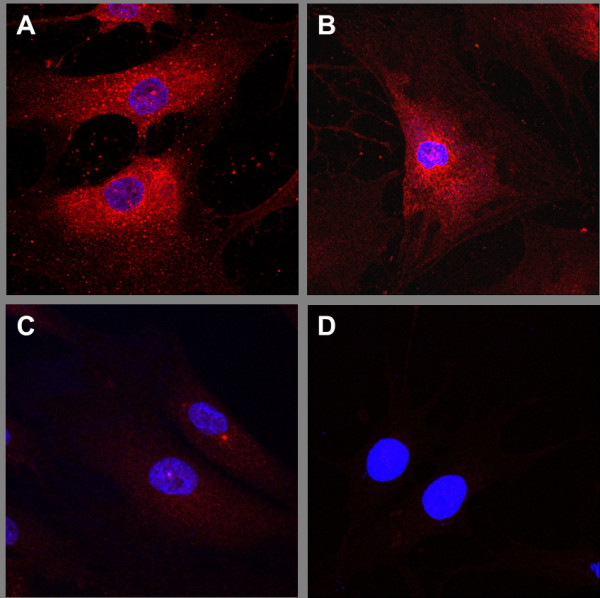
**TLR4 and IL-1RI are expressed on synovial fibroblasts**. Synovial fibroblasts were cultured in chamber slides without exogenous stimulation. TLR4 and IL-1RI expression was determined by immunocytochemical staining (red Alexa Fluor^© ^594) and nuclei were counterstained with Hoechst (blue). **A**) TLR4 staining, **B**) IL-1RI staining, **C**) staining with TLR4 specific antibody pre-incubated with blocking peptide, **D**) control staining with irrelevant rabbit IgG.

TLR4 and IL-1RI, the reciprocal signalling receptors for the HMGB1 complex partner molecules LPS, IL-1α and IL-1β, were expressed on synovial fibroblasts from both RA (RASFs) and OA (OASFs) patients as demonstrated by immunofluoresencent staining. A strong expression of both TLR4 and IL-1RI was recorded (Figure 1).

### HMGB1 in complex with LPS increases the secretion of proinflammatory cytokines from synovial fibroblasts

**Figure 2:**
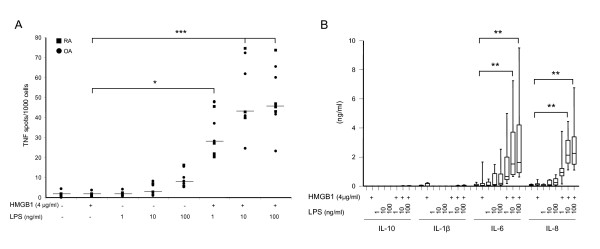
**HMGB1 in complex with LPS stimulates RASFs and OASFs to TNF, IL-6 and IL-8 production**. Synovial fibroblasts were stimulated for nine hours with **A**) HMGB1, LPS or HMGB-LPS with the indicated concentrations. The addition of HMGB1-LPS complex to cells induced a 1 to 2 log-fold increased number of TNF producing cells recorded by Elispot. Individual results from RA (squares) and OA (dots) represent results from each donor; the horizontal line indicates the median values. Significant differences were evident between HMGB1-LPS complex stimulation compared to HMGB1 simulation alone. **B**) The ability of HMGB1-LPS complexes to induce an enhanced production of IL-10, IL-1β, IL-6 and IL-8 in RASFs and OASFs was analyzed by CBA after 24 hours stimulation. HMGB1-LPS complexes at indicated concentrations induced a significantly enhanced production of IL-6 and IL-8 compared to HMGB1 stimulation alone whereas no production of IL-10 or IL-1β could be detected. Pooled data from RAFSs and OASFs where the horizontal line indicates the median values. RASF *n *= 4, OASF *n *= 5. *P-*values were calculated using Kruskal-Wallis non-parametric ANOVA test. * (*P *< 0.05) ** (*P *< 0.01) *** (*P *< 0.001).

Cultures of RASFs and OASFs were stimulated with HMGB1, LPS or complexes of HMGB1 and LPS, and the resultant cytokine production was analysed using Elispot and CBA. Stimulation with 4 μg/ml HMGB1 did not induce TNF production in cultures of RASFs or OASFs. The selected doses, 1 to 100 ng/ml of LPS did not induce any or only minor TNF production above background levels. In contrast, significant TNF production occurred when RASF or OASF were stimulated with HMGB1 preincubated with 1 to 100 ng/ml LPS as compared to HMGB1 or LPS alone (Figure 2a). To define whether the enhancement of TNF production was an isolated effect or if HMGB1-LPS complex stimulation affected the production of additional cytokines we also analyzed the production of IL-10, IL-1β, IL-6 and IL-8 using CBA. Similarly to the induced TNF production, HMGB1 in complex with LPS synergistically increased IL-6 and IL-8 production from both RASF and OASFs in a dose-dependent manner (Figure 2b). The synergistic effects of the complexes were statistically significant with a 5 to 15 and 10- to 20-fold increase in IL-6 and IL-8 production, respectively, as compared to 100 ng/ml LPS stimulation alone. Confirming the previously reported necessity of a preformed complex formation between HMGB1 and LPS, simultaneous addition of HMGB1 and LPS to cell cultures did not result in enhanced cytokine production (data not shown).

No induction of IL-10 or IL-1β production could be detected after 24 h of stimulation with HMGB1 alone, LPS alone or HMGB1 in complex with LPS. As the cytokine response detected by Elispot or CBA did not differ significantly between RASFs and OASFs, median values of recorded data from these experiments are indicated with horizontal line in Figure 2a, b.

Thus, similarly to results previously demonstrated using human peripheral blood mononuclear cells (PBMCs) [18], RASFs and OASFs respond to HMGB1 in complex with LPS by an enhanced cytokine production. OK

### HMGB1 in complex with IL-1β increases proinflammatory cytokine secretion from synovial fibroblasts

**Figure 3:**
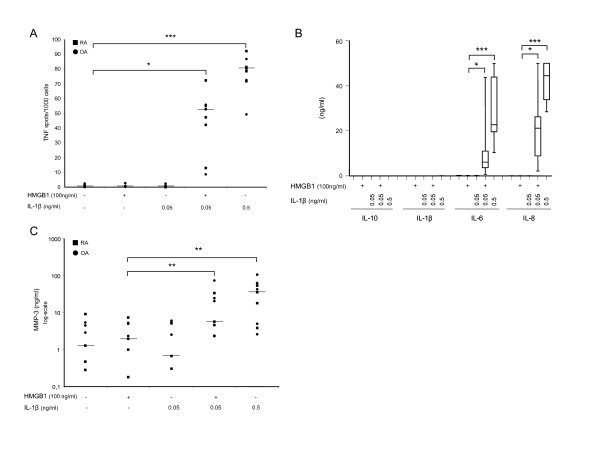
**HMGB1 in complex with IL-1β stimulates SFs to TNF, IL6, IL-8 and MMP-3 production**. Synovial fibroblasts were stimulated for nine hours with **A**) HMGB1, IL-1β or HMGB1-IL-1β complexes. Addition of HMGB1-IL-1β complexes stimulates RASFs and OASFs to a 1 to 2 log-fold increased number of TNF producing cells compared to HMGB1 simulation alone. Squares (RA) and dots (OA) represent results from each donor; the horizontal line indicates the median values. **B**) HMGB1-IL-1β complexes at indicted concentrations induced a significantly enhanced production of IL-6 and IL-8 after 24 hours of stimulation compared to HMGB1 simulation alone whereas no production of IL-10 or IL-1β could be detected. Pooled data from RAFSs and OASFs where the horizontal line indicates the median values. **C**) Enhanced MMP-3 secretion was evident with HMGB1-IL-1β stimulation after 24 hours of stimulation as recorded by ELISA. Irrespective of the level of spontaneous MMP-3 production, stimulation with HMGB1-IL-1β complex enhanced the production in 8/9 cell lines compared to HMGB1 simulation alone. Squares (RA) and dots (OA) represent results from each donor, horizontal line indicates the median values. RASF *n *= 4, OASF *n *= 5. *P-*values were calculated using Kruskal-Wallis nonparametric ANOVA test. * (*P *< 0.05) ** (*P *< 0.01) *** (*P *< 0.001).

Previous reports indicate that HMGB1 can interact with IL-1β through formation of complexes with enhanced stimulatory capacity [17,18], which is of interest regarding arthritis pathogenesis as both IL-1α and IL-1β are abundant proinflammatory cytokines in the RA arthritic joint and they have also been detected in OA joints [38,39]. RASFs and OASFs responded to IL-1β stimulation alone using a high IL-1β dose of 0.5 ng/ml. In contrast, when using a physiologically more relevant IL-1β dose of 0.05 ng/ml synovial fibroblasts did not produce cytokines. In accordance with the enhancing effects of HMGB1 in complex with LPS, preformed complexes of HMGB1 and the suboptimal dose of IL-1β induced a significant production of TNF (Figure 3a), and also of IL-6 and IL-8 (Figure 3b). The IL-6 production was increased 30- to 180-fold and IL-8 production by 100- to > 400-fold when stimulated with HMGB1-IL-1β complexes compared to stimulation with the suboptimal IL-1β concentration alone. No effect on the production of IL-10 or IL-1β could be detected when complexes were applied. Compared to the HMGB1-LPS complex experiments, the dose of HMGB1 used was much lower, 100 ng/ml, in this experimental setting, demonstrating that low, cytokine-like levels of HMGB1 display a potentiating effect on cytokine production. As the cytokine response detected by Elispot or CBA did not differ significantly between RASFs and OASFs, median values of pooled recorded data from these experiments are indicated with horizontal line in Figure 2a, b.

HMGB1-IL-1β complex stimulation induced higher cytokine levels than HMGB1-LPS complex stimulation and, correspondingly, high dose IL-1β alone was more potent in inducing cytokine production than was high dose LPS alone (Figures 2a, b and 3a, b). Furthermore, simultaneous addition of both HMGB1 and the suboptimal dose of IL-1β (without complex formation) to cell cultures did not raise cytokine production above background levels (data not shown), underlining the importance of complex formation between HMGB1 and IL-1β.

### Enhanced MMP-3 production following stimulation with complexes of HMGB1 and IL-1β

Destructive features of arthritis are partly due to the production of MMPs with the ability to degrade extracellular matrix and cartilage. We investigated whether production of MMP-3, a cartilage-degrading MMP, could be enhanced in RASFs and OASFs by stimulation with HMGB1-IL-1β complexes.

Both RASFs and OASFs spontaneously released MMP-3. Despite the differences in spontaneous MMP-3 production all but one cell line responded with significantly enhanced MMP-3 production following stimulation with HMGB1 in complex with IL-1β (Figure 3c). Enhanced MMP-3 production was observed in the non-responding cell line when stimulated with HMGB1 in complex with a higher dose of IL-1β (0.5 ng/ml), data not included in Figure 3c. This could suggest that the enhancing potential of HMGB1 in complex with IL-1β is dependent on the response to IL-1β as the ligand.

### HMGB1-LPS complexes utilise TLR4 signalling for induction of cytokine production

In order to elucidate the receptor dependence of the cytokine-enhancing effects of the investigated HMGB1-ligand complexes we investigated TLR4 requirement for HMGB1-LPS mediated cytokine production. RASFs and OASFs were incubated with detoxified LPS (LPS with the fatty acid moieties of the lipid A portion removed, resulting in a TLR4-binding LPS with 10,000-fold lower toxicity than regular LPS) for 1 to 2 h followed by stimulation with HMGB1 in complex with LPS.

**Figure 4:**
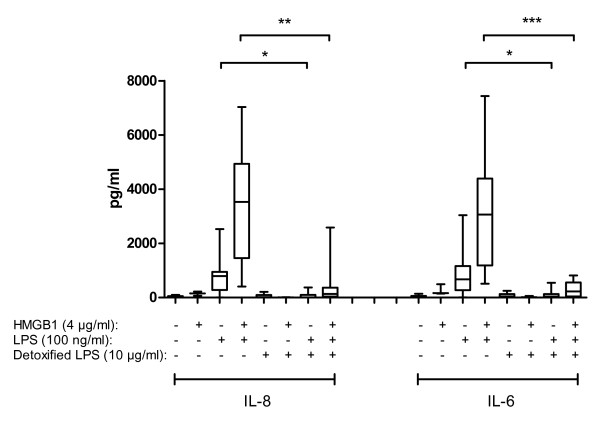
**HMGB1 in complex with LPS utilizes TLR4 for the induction of cytokine production**. Synovial fibroblasts were pretreated with detoxified LPS for one to two hours prior to the indicated stimulations. After 24 hours of stimulation, production of IL-8 and IL-6 were determined by CBA. Detoxified LPS blocked the induction of IL-8 and IL-6 production from HMGB1-LPS complex- stimulated synovial fibroblasts. Pooled data from RAFSs and OASFs where the horizontal line indicates the median values. SF *n *= 4. *P-*values were calculated using Mann Whitney test. * (*P *< 0.05) ** (*P *< 0.01) *** (*P *< 0.001).

Detoxified LPS inhibited HMGB1-LPS complex-mediated IL-6 and IL-8 production from RASFs and OASFs (Figure 4), thus demonstrating a TLR4 dependency for the cytokine-inducing signalling events induced by HMGB1 in complex with LPS. Similarly, pre-incubation with detoxified LPS inhibited the low cytokine production induced by stimulation with LPS alone (Figure 4).

### HMGB1-IL-1α and HMGB1-IL-1β complexes utilise IL-1RI signalling for induction of cytokine production

**Figure 5:**
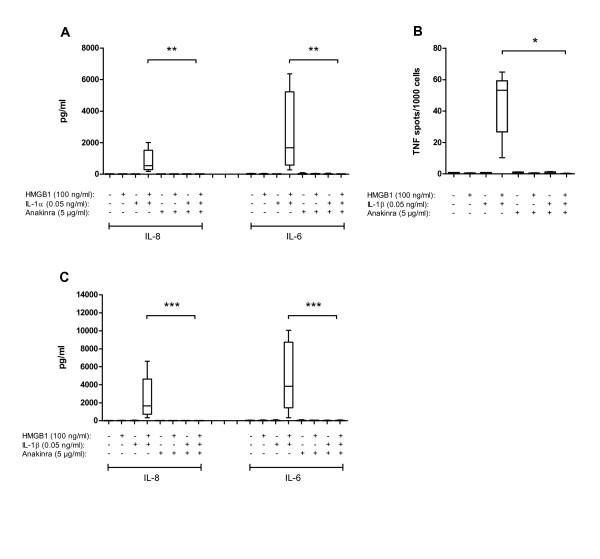
**HMGB1 in complex with IL-1α or IL-1β utilizes IL-1RI for the induction of cytokine production**. Synovial fibroblasts were pre-incubated with soluble IL-1RA one to two hours prior to indicated stimulation. IL-1RA significantly inhibited the: **A**) HMGB1-IL-1α complex mediated IL-8 and IL-6 production compared with untreated groups, determined by CBA (pooled data from RASFs and OASFs *n *= 6), **B**) HMGB1-IL-1β mediated TNF production, compared with untreated group, determined with Elispot (pooled data from RASFs and OASFs *n *= 4) and **C**) HMGB1-IL-1β complex-mediated IL-8 and IL-6 production compared with untreated groups, determined by CBA (pooled data from RASFs and OASFs *n *= 9). *P*-values were calculated using Mann Whitney test. * (*P *< 0.05) ** (*P *< 0.01) *** (*P *< 0.001).

Similar to complexes of HMGB1-IL-1β, complexes of HMGB1 with IL-1α stimulated RASFs and OASFs to significantly increased production of IL-8 and IL-6 determined by CBA, as compared to IL-1α alone (Figure 5a).

In order to investigate the role of the signalling IL-1 receptor, IL-1RI, for the observed cytokine production induced by HMGB1 in complex with IL-1α or IL-1β we utilised IL-1RA, Anakinra. RASFs and OASFs were incubated with IL-1RA for 1 h prior to stimulation with HMGB1, IL-1α, IL-1β and HMGB1 in complex with either IL-1α or IL-1β. IL-1RA significantly inhibited HMGB1-IL-1α complex mediated IL-6 and IL-8 production from RASFs and OASFs (Figure 5a) and HMGB1-IL-1β complex-mediated TNF (Figure 5b), IL-6 and IL-8 (Figure 5c) production from RASFs and OASFs. Our results indicate that IL-1RI serves as a signalling receptor for HMGB1-IL-1α- and HMGB1-IL-1β complex-mediated cytokine production.

### HMGB1-IL-1β-complexes do not utilise TLR4 signalling for induction of cytokine production

**Figure 6:**
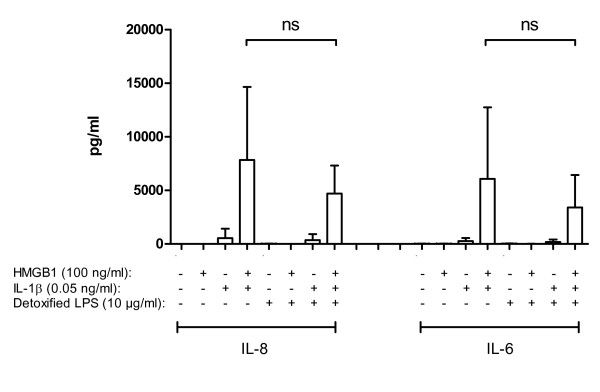
**HMGB1-IL-1β complexes do not utilise TLR4 signalling for induction of cytokine production**. Synovial fibroblasts were incubated with detoxified LPS one to two hours prior to stimulation with HMGB1-IL-1β complexes. Detoxified LPS did not inhibit the HMGB1-IL-1β complex-mediated cytokine production (pooled data SF *n *= 4). Data were analysed using Mann Whitney test.

HMGB1 has been demonstrated to interact with TLR4 and thereby to induce cytokine production [13,46]. Although the HMGB1 used in this study did not express any cytokine-inducing ability *per se*, we wanted to ascertain that the enhancing effects of the HMGB1-IL-1β complex were not due to an interaction of HMGB1 with TLR4. RASFs and OASFs were incubated with detoxified LPS 1 to 2 h prior to stimulation with HMGB1 alone or HMGB1-IL-1β-complexes and cytokine production was recorded. No significant reduction of the HMGB1-IL-1β complex-induced IL-6 and IL-8 production could be recorded as a consequence of pre-treatment with detoxified LPS (Figure 6). Our results thus demonstrate that the cytokine-enhancing ability of HMGB1-IL-1β complexes is dependent on IL-1RI signalling but not on TLR4 signalling.

## Discussion

Herein we reveal a mechanism by which HMGB1 may contribute to both inflammatory and destructive processes present during arthritis. Synovial fibroblasts stimulated with HMGB1 in complex with IL-1α, IL-1β or LPS increased their cytokine production. Additionally, HMGB1-IL-1β complexes also increased MMP-3 production. Previous studies have demonstrated that HMGB1 is released from activated immune cells and from stressed synoviocytes in arthritic joints and that blockade of extracellular HMGB1 suppresses disease progression in experimental models. Here we demonstrate that HMGB1 potentiates the effects of two endogenous molecules reported to be present in arthritic joints, namely IL-1α/IL-1β and the microbial mediator LPS. The enhancing effects are caused by complex formation between HMGB1 and the partner molecules. Such immunostimulatory features of HMGB1 in complex with IL-1β and LPS have previously been reported by us and others, while the synergistic effects of HMGB1 and IL-1α are described for the first time in this study.

So, in addition to the direct cytokine-inducing effects of HMGB1 previously reported, our results suggest that the arthritogenic features of HMGB1 can also be mediated by the enhanced activity of molecules in complex with HMGB1. HMGB1 that is actively secreted by activated macrophages or passively released from necrotic cells signals via TLR4 since the TLR4-binding epitope of the HMGB1 molecule expresses its cysteine in position 106 (C106) in reduced form, which is a prerequisite for activation of this signal pathway [47,13]. The C106 may then later be oxidized in the inflammatory milieu and will lose its capacity to signal via the TLR4 complex. However, this pacified version of HMGB1 may still act as a proinflammatory molecule if the environment contains danger molecules like IL-1α, IL-1β or LPS. HMGB1 will then act as an extracellular sensor and form complexes with these molecules that will enhance subsequent cytokine production in fibroblasts and other cells.

The ligands for complex formation with HMGB1 in this study, IL-1α, IL-1β and LPS, were chosen for three reasons; *I) *we and others have previously demonstrated a cytokine-enhancing effect of such complexes in macrophages [16,17,48]; *II) *HMGB1 [23], LPS [42], IL-1α and IL-1β [38-40] have all been detected in RA and OA synovial samples; and *III) *fibroblasts are pivotal cells in arthritic inflammation that express the suggested receptors for HMGB1 [23,35,49-51] in addition to the LPS receptor TLR4 and IL-1RI [34-37].

Complexes of HMGB1 with IL-1α, IL-1β or LPS each strongly enhanced the production of TNF, IL-6 and IL-8, while the production of both IL-10 and IL-1β was not affected. It is of interest to note that fibroblasts retrieved from both OA and RA patients shared a similar ability to respond to HMGB1-complex stimulation. Previous studies have reported a difference in extracellular HMGB1 levels in RA and OA synovial fluid with HMGB1 levels being significantly higher (54.1 ± SD 73.0 ng/ml) in RA synovial fluid than in OA synovial fluid (12.0 ± SD 17.7 ng/ml [23]. Similarly, the IL-1β levels recorded in synovial fluid levels from RA patients are roughly 10 times higher than those recorded in OA patients [39]. One can thus assume that HMGB1-IL-1β complexes are more likely formed *in vivo *during RA than during OA. This could affect the activation status of synovial fibroblasts contributing to a more inflammatory and destructive disease course in RA than in OA.

The amounts of HMGB1 and IL-1β used in our study correspond to levels recorded in RA synovial fluid; ranging from 10 to 300 ng/ml and 5 to 193 pg/ml, respectively [23,40,52]. A study by Garcia-Arnandis *et al*. [25] demonstrated that simultaneous addition of HMGB1 and IL-1β induced enhanced IL-6 and IL-8 production in OA synovial fibroblast cultures, which we did not observe in our study. However, the IL-1β concentration used in their experiments was 20-fold higher than in our experimental setup, and also higher than levels recorded in RA synovial fluid. It is plausible that the high IL-1β levels used could lead to HMGB1-IL-1β complex formation during the cell stimulation and thus their findings are in agreement with our results.

LPS, as well as other constituents of various pathogens, have been reported to be present in arthritic joints [41,42]. This has led to the hypothesis that infections can be both a cause of arthritis onset and also of disease exacerbation. However, no infectious agent in particular has been pinpointed to be associated with chronic arthritis. The data presented in this paper together with earlier studies on the interaction of HMGB1 with different TLR-ligands suggest that HMGB1 might be a unifying factor for the contribution of various infections to arthritis pathogenesis.

Our data clearly demonstrate the striking ability of HMGB1 complexes to enhance both cytokine production and MMP-3 production by SFs when compared to equivalent doses of the ligand molecules alone. We had originally hypothesized that the enhancing effects would be mediated by simultaneous engagement of an HMGB1 receptor (RAGE or TLR4) and the partner ligand receptor. By blocking IL-1RI and TLR4 with the respective receptor antagonists (IL-1 receptor antagonist or detoxified LPS) we could demonstrate that the stimulatory activities of the HMGB1-IL-1α and IL-1β complexes were mediated via the IL-1RI and that the stimulatory activity of the HMGB1-LPS complex was mediated via TLR4. Interestingly, blockade of TLR4 did not suppress the stimulation induced by HMGB1-IL-1β complexes, thus ruling out that the synergistic effects were mediated by a simultaneous interaction of TLR4 and IL-1RI. This conclusion is also supported by the fact that the HMGB1 used in our studies did not alone possess an endogenous cytokine-inducing capacity, this otherwise being mediated through TLR4 interaction [13,46]. Attempts to block RAGE, the most studied receptor for HMGB1, using a receptor antagonist failed as we could not define a functional antagonist. Results from studies when soluble RAGE (sRAGE) was added to the cell culture (data not included) demonstrated that sRAGE could suppress the activity of the HMGB1 complexes. However, this only confirms that HMGB1 can bind to RAGE; the suppressive effects were most likely caused by steric hindrance rather than by an inactivation of RAGE signalling. Data from our laboratory (H. Hreggvidsdottir *et al*., submitted manuscript) indicate that RAGE is not involved in HMGB1 complex signaling as macrophages from RAGE-deficient mice respond equally well to HMGB1 complex stimulation as from wild type mice. However, a remaining possibility for the mechanism of HMGB1 complex-induced enhancement could be the involvement of an as yet undefined HMGB1 receptor in a receptor-pair with the partner ligand receptor. A second possibility could be a multiaggregation of ligand receptors caused by the HMGB1-ligand complex leading to enhanced activity. Both scenarios deserve further investigations.

## Conclusions

Preformed complexes of HMGB1 with IL-1α, IL-1β or LPS have the ability to strongly enhance production of both proinflammatory mediators and of tissue destructive enzyme by synovial fibroblasts derived from RA and OA patients. HMGB1 thus acts as an endogenous amplifier endowed with a capacity to magnify responses to trace amounts of endogenous and exogenous danger signals. This effect is mediated via the reciprocal ligand receptors, IL-1RI and TLR4, for the ligands complexed to HMGB1 investigated in this study. HMGB1 without direct cytokine-inducing effects on its own might be present in arthritic joints as HMGB1 is released by apoptotic cells. Furthermore, exposure of cytokine-inducing, reduced HMGB1 to an oxidative burst during inflammation may downregulate its direct proinflammatory features by changing its redox status. We demonstrate that non-cytokine-inducing HMGB1 can form strongly inflammation-enhancing complexes with inflammatory mediators present in arthritic joints. These HMGB1 complexes act on both synovial fibroblasts and on monocytes and enhance their activation status. Thus in addition to the direct cytokine-inducing effect of HMGB1 previously described, we, herein, demonstrate a second mechanism by which HMGB1 may contribute to the arthritogenic process.

Through this study we have increased knowledge of the proinflammatory functions of HMGB1 in arthritis in both RA and OA settings. We have demonstrated enhancing effects of HMGB1 on both inflammatory and destructive disease mechanisms and further consolidated HMGB1 as a putative target for successful therapy.

## Abbreviations

CpG-DNA: short single-stranded synthetic DNA molecules that contain a cytosine followed by a guanine; Elispot: enzyme-linked immunospot assay; HMGB1: high mobility group box protein 1; IL-1α: interleukin 1 alpha; IL-1β: interleukin 1 beta; IL-1RI: interleukin 1 receptor type 1; IL-1RA: interleukin 1 receptor antagonist; LPS: lipopolysaccharide; MMP-3: matrix metalloproteinase 3; OA: osteoarthritis; OASF: osteoarthritis synovial fibroblasts; PBMCs: peripheral blood mononuclear cells; RA: rheumatoid arthritis; RAGE: receptor for advanced glycated end products; RASF: rheumatoid arthritis synovial fibroblasts; TMB: tetramethylbenzidine; TNF: tumour necrosis factor; TLR: Toll-like receptor.

## Competing interests

The authors declare that they have no competing interests.

## Authors' contributions

HW was responsible for the study design, experimental work, data collection and the manuscript preparation. HS participated in study design, experimental work and statistical analysis and in manuscript preparation. HH participated in study design, experimental work and in manuscript preparation. KP performed the immunocytochemical stainings and ACA participated in cell culture work and with technical support during many experiments. UA was responsible for study design, supervision and manuscript preparation. HEH was responsible for study design, supervision and she drafted the manuscript. All authors read and approved the final manuscript.
